# The Veterinary School at Milan, Italy*Wochenschrift für Thierheilkunde, No. 24, 1884.

**Published:** 1884-10

**Authors:** 


### EDITORIAL NOTES.

THE VETERINARY SCHOOL AT MILAN, ITALY.*—Prof. Dr. N. Lanzittotti Buonsanti, Director of this school, has just published its history in a brochure of 188 pages, which is a valuable contribution to historical veterinary literature.

He considers his subject in six periods, the first, commencing in 1769 and ending in 1790, includes the foundation of the Veterinary Schools in France. The second period marks the foundation of the school at Milan on the first of February, 1791, and extended to 1807, in which the necessary buildings were erected for the students and teachers as well as hospital. The first professors were Valpi and Lucchini. Between 1808 and 1834, the school was organized after those of France through the power of Napoleon, who had Italy under his thumb at that time. The fourth period, 1834 to 1858, saw a reform in the school which, under Austrian rule, was modeled after that at Vienna. The fifth period, 1859 and 1860, again marked a revision in the school corresponding to certain changes in Austria. Towards the end of 1860 it again came under Italian control, and became an independent institution, having the rank of a University, being under the supervision of the Ministry for Public Education. The course is four years, the Diploma, Doctor of Veterinary Medicine.

For further details we must refer to the original.

* Wochenschrift für Thierheilkunde, No. 24, 1884.

